# Uncovering latent profiles of multidimensional work attitudes: cross-cultural evidence from Poland and Vietnam

**DOI:** 10.3389/fpsyg.2026.1749901

**Published:** 2026-05-28

**Authors:** Paweł Jurek, An-Nhan Thi Lac, Bao-Tram Ngoc Le, Michał Olech, Joanna Różycka-Tran

**Affiliations:** 1 Institute of Psychology, University of Gdańsk, Gdańsk, Poland; 2 Department of Psychology, Medical University of Gdańsk, Gdańsk, Poland; 3 Faculty of Psychology, University of Social Sciences and Humanities, Vietnam National University, Ho Chi Minh City, Vietnam

**Keywords:** cross-cultural comparison, job satisfaction, latent profile analysis, Poland, Vietnam, work attitudes, work engagement

## Abstract

**Background:**

Employees’ relationships with work are multidimensional (affective, cognitive–evaluative, and motivational–behavioral) yet variable-centered studies can mask patterned heterogeneity. We integrated these strands and asked: Which naturally occurring constellations of work attitudes emerge among employees, and are they similar across Poland and Vietnam?

**Methods:**

A cross-sectional survey of Polish (*N* = 358) and Vietnamese (*N* = 305) employees assessed positive/negative organizational affect, four job-satisfaction facets (development, salary, tasks, relationships), work engagement, and working excessively/compulsively. We used: Multigroup Confirmatory Factor Analysis (MGCFA) to established measurement invariance of the tools across countries, Latent Profile Analysis (LPA) to identified profiles in the pooled sample, multigroup LPA to test cross-national profile similarity. Profile prevalence and links with gender, tenure, position, and sector were examined.

**Results:**

A three-profile solution best balanced fit and parsimony: (1) Moderately Disengaged; (2) Overworked & Discontent; (3) Engaged & Satisfied. Configural similarity held across countries, but means and dispersions differed. Engaged & Satisfied was more prevalent in Vietnam (80%) than Poland (57%), whereas Moderately Disengaged was more common in Poland (36%) than Vietnam (11%). Gender and sector were unrelated to profile membership; managers were overrepresented in Engaged & Satisfied; this group also had longer tenure.

**Conclusion:**

Distinct, cross-culturally recurring configurations of work attitudes align with JD–R, Affective Events, and Self-Determination frameworks. Tailored interventions should target development, task design, and recognition to elevate Moderately Disengaged employees, and workload/recovery and social-climate improvements for the Overworked & Discontent group. Cross-national differences in levels and prevalence underscore the need for contextual adaptation of organizational policies.

## 1 Introduction

A growing body of organizational research recognizes that employees’ relationships with work are multifaceted, encompassing affective orientations toward the organization (e.g., Ashkanasy and Dorris, 2017; Judge et al., 2017), cognitive–evaluative judgments about key job domains (e.g., Parker and Knight, 2024), and motivational–behavioral investment in work (e.g., Bakker et al., 2023; Mazzetti et al., 2023; Olafsen and Deci, 2020). Classic variable-centered approaches typically estimate average associations among these constructs, implicitly assuming a single homogeneous population. Yet employees frequently combine these elements in distinct configurations, for example, pairing strong engagement with moderate overwork, or reporting high task satisfaction alongside weak affective attachment to the employer. Such patterned heterogeneity can be obscured by variable-centered models. Responding to this gap, person-centered methods, particularly Latent Profile Analysis (LPA), enable the recovery of naturally occurring constellations of work attitudes within the workforce (Wang and Hanges, 2011; Morin and Marsh, 2015; Oberski, 2016).

The present study advances this agenda in three ways. First, we propose a multidimensional work attitudes framework integrating: (a) positive and negative affective orientations toward the organization, (b) cognitive–evaluative judgments: satisfaction with professional development, salary, tasks, and workplace relationships, and (c) motivational–behavioral orientations spanning work engagement versus working excessively and compulsively. Second, we adopt a person-centered LPA to identify latent profiles that reflect qualitatively and quantitatively distinct configurations of these dimensions, thereby illuminating both adaptive (e.g., engaged–satisfied) and maladaptive (e.g., excessive and compulsive overwork) patterns that co-exist in organizations (Morin et al., 2018; Petitta et al., 2025). Third, we take a cross-cultural perspective by comparing Poland and Vietnam: two contexts that differ in socio-cultural norms around work, and test profile similarity across groups, evaluating configural, structural, dispersion, and distributional similarities (Eid et al., 2003; Woo et al., 2024).

Grounded in the Job Demands–Resources model (Bakker and Demerouti, 2017), Affective Events Theory (Weiss and Cropanzano, 1996), and Self-Determination Theory (Deci and Ryan, 2000), we argue that employees’ work attitudes emerge from the interplay of job demands and resources, cumulative affective experiences, and the satisfaction or frustration of basic psychological needs, yielding distinctive attitudinal configurations. Whereas prior research has often examined these strands in isolation, their integration within a person-centered, cross-cultural design remains rare. Our study addresses this gap by: (a) mapping profiles of multidimensional work attitudes, (b) testing their similarity across national groups, and (c) laying the groundwork to examine how these profiles relate to sociodemographic variables.

## 2 Theoretical framework

### 2.1 Multidimensional work attitudes: affective, cognitive–evaluative, and motivational–behavioral components

Our framework synthesizes three complementary components: (1) affective orientations toward the organization, (2) cognitive–evaluative judgments, and (3) motivational–behavioral orientations.

Positive affective orientation toward the organization (PosAtt) and negative affective orientation toward the organization (NegAtt) reflect employees’ generalized affective responses to their long-term participation in organizational life. These orientations develop through repeated experiences of positive or negative emotions associated with work conditions and task performance over time (Jurek and Adamska, 2017). Drawing on Affective Events Theory (Weiss and Cropanzano, 1996; see also Elfenbein, 2023), repeated exposure to negative work events and stressors elicits adverse emotional reactions that, when experienced frequently, consolidate into negative attitudes toward the organization, often contributing to burnout. In contrast, the accumulation of positive emotional experiences in the workplace fosters the development of positive affective orientations, which enhance work engagement and strengthen organizational identification. Employees with high levels of PosAtt tend to associate their workplace with pleasant emotions, enthusiasm, and pride in belonging to the organization. Conversely, those high in NegAtt perceive their workplace as emotionally discomforting and tend to associate it with feelings such as shame or alienation, reflecting a lack of identification with the employer. Research further suggests that positive affective experiences can buffer the impact of negative or stressful events, particularly when the two are similar in nature (Ohly and Schmitt, 2015). In this research, PosAtt and NegAtt are treated as key affective indicators for identifying latent employee profiles characterized by distinct patterns of organizational attitudes.

Building upon affective orientations, employees’ overall work-related attitudes can also be understood from a cognitive–evaluative perspective, reflecting their judgments of specific aspects of the employment experience. Four such dimensions are included in this study: satisfaction with professional development opportunities (SatDev), satisfaction with salary (SatSal), satisfaction with tasks (SatTas), and satisfaction with workplace relationships (SatRel).

Satisfaction with professional development opportunities (SatDev) refers to employees’ evaluations of the extent to which their work environment enables both vertical career progression and continuous skill enhancement. Professional development encompasses advancement to higher-level positions and the ongoing acquisition of competencies that foster self-realization and employability. Higher satisfaction with development opportunities is associated with greater initiative, stronger organizational commitment, lower absenteeism, and greater openness to change (Juchnowicz et al., 2018).

Satisfaction with salary (SatSal) captures employees’ perceived fairness and adequacy of their financial compensation and benefits. Pay satisfaction is closely related to distributive and procedural justice – concerning the fairness of reward allocation and the fairness of organizational policies and procedures governing pay systems. Consistency between pay level, self-assessed competence, and individual performance is a key determinant of high salary satisfaction (Jurek and Olech, 2019).

Satisfaction with tasks (SatTas) pertains to employees’ evaluations of the nature and content of their work, including task variety, complexity, and perceived meaningfulness. According to Job Characteristic Theory (Oldham, 2017), meaningful and engaging tasks promote intrinsic motivation, job satisfaction, and performance quality. Recognizing the significance of one’s work has been identified as a core psychological state linked to greater well-being and engagement (Van Wingerden and van der Stoep, 2018).

Satisfaction with workplace relationships (SatRel) concerns employees’ perceptions of the quality of their social interactions and support within the workplace. Positive relationships with colleagues and supervisors are key determinants of job satisfaction and team climate (Chou and Robert, 2008; Ducharme and Martin, 2000). Supportive and fair managerial behavior further strengthens employees’ satisfaction and organizational commitment (Koh and Boo, 2004).

Together, these four satisfaction domains represent the cognitive–evaluative components of the Multidimensional Work Attitudes model, complementing affective orientations toward the organization to provide a comprehensive view of employees’ psychological relationships with their work.

Within the Multidimensional Work Attitudes framework, work engagement (EngWork), working excessively (ExcWork), and working compulsively (ComWork) represent motivational–behavioral orientations that capture nuanced differences in employees’ relationships with their job. Work engagement is defined as a positive, fulfilling, work-related state of mind characterized by vigor, dedication, and absorption (Knight et al., 2017). It reflects an active investment of energy and effort, accompanied by enthusiasm and psychological involvement in work tasks (Saks, 2006). As a positively valued work attitude, engagement is consistently linked to enhanced job performance, organizational citizenship behaviors, organizational commitment, job satisfaction, and retention intentions (Christian et al., 2011). Engaged employees experience their work as meaningful and are supported by positive job resources such as autonomy, opportunities for professional growth, social support, and recognition (Mazzetti et al., 2023).

In contrast, ExcWork and ComWork capture maladaptive work-related tendencies associated with workaholism. According to Schaufeli et al.’ (2009) conceptualization, workaholism is defined as an irresistible inner drive to work extremely hard, comprising two distinct yet related dimensions: a behavioral component (working excessively) and a cognitive component (working compulsively). ExcWork reflects the behavioral tendency to devote disproportionate time and energy to work, often beyond what is reasonably required, while ComWork denotes an internal compulsion and obsessive thinking about work, even in nonwork contexts. Although both constructs indicate high involvement in work, they are qualitatively different from engagement: engagement represents energetic and self-endorsed involvement, whereas ExcWork and ComWork stem from an uncontrollable inner pressure rather than intrinsic motivation. Empirical research consistently demonstrates that workaholism (particularly ComWork) is negatively associated with engagement (Schaufeli et al., 2008) and performance (Shimazu et al., 2015), while being positively related to burnout and psychological distress (Balducci et al., 2021).

In this research, EngWork, ExcWork, and ComWork are conceptualized as interrelated components forming a continuum from adaptive, fulfilling engagement to maladaptive, compulsive overwork. This approach allows for a more nuanced understanding of how employees differ in their motivational and behavioral orientations toward work.

Taken together, these constructs provide a comprehensive representation of employee work-related functioning, spanning motivational effort, emotional experience, and evaluative judgment. Integrating them within a person-centered framework enables the identification of distinct employee subgroups that combine these dimensions in meaningful ways, thereby offering insights into both adaptive and maladaptive patterns of workplace functioning.

Understanding how employees relate to their work requires an integrative perspective that captures the complexity of their emotional, cognitive, and motivational orientations. Contemporary frameworks in work and organizational psychology such as: the Job Demands–Resources (JD–R) model (Bakker and Demerouti, 2017), Affective Events Theory (Weiss and Cropanzano, 1996; Elfenbein, 2023), and Self-Determination Theory (Deci and Ryan, 2000), jointly emphasize that employees’ attitudes emerge from the dynamic interplay between individual needs, affective experiences, and contextual conditions. The JD–R model distinguishes between job demands that may lead to strain or burnout and job resources that foster engagement and well-being, while Affective Events Theory highlights how cumulative emotional experiences at work shape enduring affective orientations toward the organization. Self-Determination Theory further explains how the satisfaction or frustration of basic psychological needs drives autonomous or controlled forms of work motivation.

### 2.2 Demographic correlates of work attitudes profiles

In addition to cross-national differences, the present study examines whether membership in latent work attitude profiles is systematically associated with selected sociodemographic characteristics, including gender, job seniority, job position, and employment sector. These variables are included as theoretically meaningful correlates, as prior research indicates that individual and structural characteristics are linked to variation in employees’ attitudes and work-related behaviors (Rudolph et al., 2017).

Job position and seniority are particularly relevant in this context, as they reflect employees’ location within organizational hierarchies and their accumulated work experience. Higher-level positions are typically associated with greater autonomy, access to resources, and decision-making authority, which are positively related to engagement and job satisfaction (Humphrey et al., 2007). Accordingly, employees in managerial roles and with longer tenure may be more likely to display more adaptive configurations of work attitudes.

Additionally, job seniority may be associated with increased role clarity, organizational embeddedness, and greater stability of work attitudes. Although direct evidence on tenure is mixed, meta-analytic findings indicate that employee age is positively related to more favorable and stable job attitudes (Ng and Feldman, 2010), which is closely related with job seniority in organizational contexts.

Gender is included as a key individual characteristic that may relate to differences in work experiences and attitudes due to socially structured roles and differential exposure to job demands and resources. Research on self-censorship suggests that women are more likely than men to withhold information about observed irregularities, particularly when they perceive that their voice will not be heard, and this tendency persists even in managerial positions (Adamska et al., 2022). At the same time, organizational factors such as procedural justice and a communal climate can reduce self-censorship, especially among women, indicating that gender may shape both work behaviors and underlying organizational attitudes.

Employment sector (public vs. private) captures differences in institutional and organizational environments, which have been shown to be associated with employees’ attitudes and behavior. Public sector employment is often characterized by higher job security and procedural stability, whereas private sector contexts may involve stronger performance pressures and market-driven incentives, potentially leading to different configurations of satisfaction, engagement, and strain (Perry et al., 2010).

In the present study, these sociodemographic variables are examined as correlates to assess whether the distribution of latent profiles differs across employee groups. This approach enables the identification of systematic differences in profile prevalence (rather than mean-level differences) and provides additional insight into how multidimensional work attitudes are distributed across individuals with different demographic and occupational characteristics.

### 2.3 Person-centered approach

Following recent person-centered analyses in organizational psychology (e.g., Petitta et al., 2025), Latent Profile Analysis (LPA) offers a powerful approach to identifying subpopulations of employees who share qualitatively and quantitatively distinct configurations of multidimensional work attitudes. Unlike variable-centered methods, which assume a single homogeneous population and estimate average relationships among constructs, LPA recognizes that employees may differ systematically in how affective, cognitive, and motivational dimensions of work attitudes co-occur within individuals (Morin and Marsh, 2015).

Since the publication of the Organizational Research Methods feature topic on latent class procedures (Wang and Hanges, 2011), person-centered methodologies such as LPA and LCA have become well established in organizational sciences. Their key heuristic advantage lies in their typological logic: they generate empirically derived profiles that reflect naturally occurring patterns of work-related orientations, enabling the identification of both adaptive and maladaptive combinations of attitudes. In practical terms, LPA classifies individuals probabilistically rather than deterministically – each person is assigned a likelihood of belonging to each latent profile, taking measurement error into account (Morin et al., 2018). This probabilistic and model-based nature enhances the precision of classification and allows for testing profile predictors and outcomes within a unified statistical framework.

The usefulness of LPA in the context of Multidimensional Work Attitudes stems from its ability to reveal subgroups of employees who combine positive affective orientations (e.g., engagement, satisfaction) and maladaptive patterns (e.g., compulsive or excessive work) in distinct ways. However, applying LPA meaningfully requires several methodological and theoretical conditions to be met. First, the selected indicators should represent conceptually distinct yet theoretically related dimensions (e.g., affective, cognitive–evaluative, and motivational–behavioral attitudes) that jointly capture the multidimensional construct. Second, adequate sample size is essential to ensure the stability and replicability of the extracted profiles. Third, the model should be grounded in theory, allowing for meaningful interpretation of the emergent profiles rather than purely data-driven segmentation (Morin and Marsh, 2015; Morin et al., 2018). When these criteria are satisfied, LPA provides a robust tool for uncovering the complex and nuanced ways in which employees experience and interpret their work environment. The equations should be inserted in editable format from the equation editor.

### 2.4 Cross-cultural profile similarity

In cross-cultural organizational research, LPA has increasingly been used to uncover subpopulations of employees whose work-related attitudes or experiences differentially cohere within and across national contexts. Extending this framework cross-culturally, we examine whether similar latent profiles of work attitudes emerge across institutional and cultural contexts (Wang and Hanges, 2011; Woo et al., 2024). For example, a profile of strong organizational commitment and engagement that emerges in one country may combine with moderate levels of workaholic tendencies in another, or even split into more nuanced subgroups, reflecting cultural norms around work, autonomy, collectivism, and risk.

One of the primary advantages of LPA in a cross-cultural design is its capacity to test profile similarity (i.e., measurement invariance) across groups via multigroup LPA. Through such comparisons, researchers can assess whether the latent typologies themselves are structurally equivalent (same number of profiles, same shape) or whether profile sizes, means, or indicator variances differ by country (Eid et al., 2003; Woo et al., 2024). This enables statements not only about “global” profiles but also about which profiles are universal (etic) versus culturally specific (emic), and how cultural context may moderate the meaning or prevalence of given attitude patterns.

### 2.5 Comparative institutional context: Poland and Vietnam

Although Poland and Vietnam differ in cultural, governance, and economic institutions, both countries represent rapidly developing economies that have undergone profound social and labor transformations in the past three decades. Drawing on the institutional hierarchy model (North, 1990; Scott, 2019), we conceptualize national contexts as layered systems in which cultural, socio-political-legal, and economic institutions jointly shape employees’ work experiences and attitudes.

According to Hofstede’s cultural dimensions (Hofstede et al., 2010; Hofstede Insights, 2023), both Poland and Vietnam display relatively high power distance (68 and 70, respectively), implying acceptance of hierarchical relationships in organizations. However, the two countries diverge markedly in individualism (Poland = 47; Vietnam = 30) and uncertainty avoidance (Poland = 93; Vietnam = 30). Polish employees operate within a culture characterized by moderate individualism and strong preferences for predictability and clear rules, whereas Vietnamese employees are embedded in a collectivist and low-uncertainty culture that values adaptability and relational harmony. Poland also scores higher on achievement orientation (64 vs. 40), while Vietnam is slightly higher on indulgence (35 vs. 29). These cultural configurations suggest that Polish employees may place greater emphasis on procedural fairness and role clarity, whereas Vietnamese employees may prioritize social relatedness and long-term relational investment in work.

Governance institutions mediate how cultural norms are translated into workplace realities (Scott, 2019). They shape rules of participation, collective voice, and social protection. In line with formal constitutional definitions, Poland is a parliamentary democracy with a social market economy regulated by tripartite dialogue among government, employers, and trade unions [Organisation for Economic Cooperation and Development (OECD), 2023]. Collective bargaining and employment protection are enshrined in the Labour Code, and social security coverage is near-universal. Vietnam is a socialist-oriented market economy under the leadership of the Communist Party of Vietnam [International Labour Organization (ILO), 2022a]. The Vietnam General Confederation of Labour (VGCL) serves as the sole officially recognized trade-union federation, and wage setting remains partly centralized.

Economic institutions constitute both an outcome and a mechanism of institutional dynamics (North, 1990). In 2020, Poland’s Gross National Income per capita (PPP) reached 35,720 international dollars, compared with 8,150 in Vietnam (World Bank, 2023). Both countries maintained low unemployment rates (Poland 3.2%; Vietnam 2.3%) and positive GDP growth (2.0 and 3.4%, respectively), reflecting economic resilience during the global downturn. Poland’s labor force was concentrated in services (59%), followed by industry (31%) and agriculture (9%), whereas Vietnam remained more agrarian, with agriculture (37%), industry (29%), and services (34%) [World Bank, 2023; International Labour Organization (ILO), 2022b]. Self-employment accounted for approximately 19.9% of Polish workers and 54.3% in Vietnam, and informal employment was about 10–14% in Poland (Eurostat, 2020) versus 56% in Vietnam [International Labour Organization (ILO), 2020]. Both economies, however, have expanded rapidly and integrated into global markets, creating rising demand for knowledge-intensive and service-sector jobs.

Understanding work attitudes in Poland and Vietnam thus requires situating them within these layered institutional contexts. Below, we summarize cultural, governance, and economic factors relevant to interpreting potential profile differences and connect them to our person-centered approach.

The integrative model proposes that cultural norms (via national value dimensions) shape baseline motivational and cognitive frameworks (e.g., emphasis on achievement, autonomy, or uncertainty avoidance). Socio-political-legal institutions determine the extent of employee voice and regulation, influencing perceived justice and access to resources. Economic institutions determine resource endowments and market dynamics, affecting employment security and investment in work. Variations in work attitudes across Poland and Vietnam thus reflect the interplay of these institutional layers. Employees internalize cultural norms, respond to governance structures, and act within economic constraints, resulting in distinctive constellations of work attitudes. The person-centered LPA approach employed in this study allows for empirical identification of such cross-nationally embedded profiles.

## 3 Methods

### 3.1 Participants and procedure

We conducted a cross-sectional survey among Polish (*N* = 358) and Vietnamese (*N* = 305) employees. Participants were recruited using a non-probability convenience sampling strategy. Data collection was conducted by collaborating researchers in Poland and Vietnam using organizational networks, professional contacts, and online distribution channels (e.g., email invitations). The survey was administered online in both countries using comparable procedures.

Although no formal stratified sampling approach was employed, efforts were made during recruitment to obtain a heterogeneous sample with regard to gender, age, job position, and organizational sector. This was achieved through targeted outreach to diverse organizations and professional groups. However, the sample should not be considered representative of the national employee populations, and the findings should be interpreted accordingly. Participation was voluntary and anonymous, and all respondents provided informed consent prior to completing the survey. The study was conducted in accordance with the ethical standards of the Declaration of Helsinki and approved by the Research Ethics Committee at the University of Gdansk (approval no. 31/2025/WNS).

As shown in Table 1, the total sample comprised 663 employees, of whom 38% were men in both national subsamples. The mean age was comparable across groups, averaging approximately 35 years. Polish employees reported slightly longer job tenure than Vietnamese employees. Most participants held specialist or managerial positions, and the distribution of employment areas varied between countries: employees in the Vietnamese sample were more often engaged in service-related roles, whereas participants in the Polish sample more frequently worked in production, sales, or support functions. The organizational size distribution was relatively balanced, with a predominance of employees from large enterprises. Regarding ownership, a greater proportion of participants in the Polish sample worked in the private sector, while respondents in the Vietnamese sample were more frequently employed in the public sector. Formal statistical comparisons between countries are presented in Table 1.

**Table 1:** Sample composition by country: demographic and occupational characteristics of polish and Vietnamese employees (N = 663).

Characteristic	Poland	Vietnam	Total sample	Test statistic	*p*-value
*N*	358	305	663		
Gender (Male %)	38	38	38	χ^2^ < 0.01	0.99
Age (M ± SD)	35.63 ± 10.17	34.84 ± 7.47	35.27 ± 9.03	t = 1.16	0.25
Seniority	13.76 ± 9.12	11.64 ± 7.01	12.79 ± 8.29	t = 3.38	< 0.01
Job position *n* (%)				χ^2^ = 0.12	0.94
Entry-level	68 (19)	55 (18)	123 (19)		
Specialist	120 (34)	102 (33)	222 (33)		
Managerial	170 (47)	148 (49)	318 (48)		
Area *n* (%)				χ^2^ = 65.05	< 0.01
Production or technology	60 (17)	18 (6)	78 (12)		
Sales or customer service	112 (31)	49 (16)	161 (24)		
Support	97 (27)	79 (26)	176 (27)		
Service	89 (25)	159 (52)	248 (37)		
Size *n* (%)				χ^2^ = 35.47	< 0.01
Micro	82 (23)	29 (10)	111 (17)		
Small	80 (22)	76 (25)	156 (23.5)		
Medium-sized	59 (17)	97 (32)	156 (23.5)		
Large	137 (38)	103 (33)	240 (36)		
Sector *n* (%)				χ^2^ = 12.95	< 0.01
Public	130 (36)	154 (50)	248 (43)		
Private	228 (64)	151 (50)	379 (57)		

### 3.2 Measures

Affective orientations toward the organization. We used the PNE scale (Jurek and Olech, 2019), comprising two 7-item subscales: positive affective orientation toward the organization (PosAtt; e.g., “I feel proud that I work for my organization.”) and negative affective orientation toward the organization (NegAtt; e.g., “When I’m at my workplace, I only dream of leaving it”). Items were rated on a 5-point Likert scale from 1 (strongly disagree) to 5 (strongly agree).

Cognitive–evaluative judgments. We assessed cognitive–evaluative aspects of job attitudes with the SAT-20 scale (Jurek and Olech, 2019), which covers four domains: satisfaction with professional development (SatDev: 5 items; e.g., “My organization gives me a real chance of promotion”), satisfaction with salary (SatSal: 6 items; e.g., “I receive appropriate recognition for the results of my work”), satisfaction with tasks (SatTas: 5 items; e.g., “I have an opportunity to participate in a variety of tasks which require different skills”), and satisfaction with workplace relationships (SatRel: 4 items; e.g., “I can count on help and support from my colleagues”). Participants rated their agreement on a 5-point Likert scale (1 = strongly disagree to 5 = strongly agree).

Motivational–behavioral orientations. Work engagement was assessed using the 9-item Utrecht Work Engagement Scale (UWES-9; Schaufeli et al., 2006), which captures three dimensions: vigor, dedication, and absorption. Example items include: “I am enthusiastic about my job,” “Time flies when I’m working,” and “My job inspires me.” Responses were provided on a 7-point frequency scale ranging from 0 (never) to 6 (always/every day). Working excessively and working compulsively were measured with the 10-item Dutch Work Addiction Scale (DUWAS; Schaufeli et al., 2009), consisting of five items per dimension. Example items are: “I stay busy and keep many irons in the fire,” “I feel guilty when I take time off work,” and “There’s something inside me that drives me to work hard.” Responses were given on a 4-point agreement scale from 1 (strongly disagree) to 4 (strongly agree).

In the Polish sample, the SAT-20 and PNE were administered in their original Polish versions, while the UWES and DUWAS were used in their validated Polish adaptations (available at: https://www.wilmarschaufeli.nl/tests). In the Vietnamese sample, all instruments were translated from English into Vietnamese using a forward–backward translation procedure, involving independent forward translation (English to Vietnamese) and back-translation (Vietnamese to English) by bilingual experts. The psychometric properties of the scales and their measurement invariance across language versions are presented in the Results section.

### 3.3 Analytic strategy

#### 3.3.1 Preliminary measurement checks

Before estimating the mixture models, we examined cross-national measurement equivalence of the indicators using Multigroup Confirmatory Factor Analysis (MGCFA), sequentially testing configural, metric, and scalar invariance to ensure comparability across countries. When full scalar invariance was not tenable, we adopted partial scalar invariance by freeing a minimal set of noninvariant intercepts, with all releases justified and their implications for latent mean comparisons explicitly reported. We also assessed internal consistency in each country (i.e., coefficient *ω* and *α*) to document the reliability of all measures.

#### 3.3.2 Latent profile identification (pooled sample)

We fit Gaussian mixture models to the pooled data (*N* = 663), incrementing the number of profiles (K) from 1 upward. Indicators were treated as continuous; within-class variances and covariances were freely estimated but constrained to be equal across profiles, so class separation was captured by class-specific means. Models were estimated with MLR using extensive random starts (1,000 initial/100 final), and the best log-likelihood was replicated. Model selection balanced BIC/SABIC, the parametric bootstrap LRT (BLRT), entropy, minimum class size, and substantive interpretability (Wang and Hanges, 2011; Morin and Marsh, 2015).

#### 3.3.3 Multigroup LPA: profile similarity across countries

To assess cross-national generalizability, we estimated multigroup LPA with country as a known class and tested a hierarchy of profile-similarity constraints (Eid et al., 2003; Woo et al., 2024): (a) configural similarity: same number of profiles in Poland and Vietnam, all class-specific parameters free; (b) structural (shape) similarity: equality constraints on indicator means within corresponding profiles across countries; (c) dispersion (scale) similarity: additional equality of indicator variances within profiles; (d) distributional similarity: additional equality of class proportions. Model comparisons relied on BIC/SABIC, classification quality, and interpretability. Where stricter constraints degraded fit or interpretability, we retained the least restrictive plausible model.

#### 3.3.4 Profile prevalence analysis

To examine the distribution of work attitude profiles across countries and sociodemographic groups, we conducted a series of frequency and association analyses. First, the prevalence of each latent profile was computed separately for Polish and Vietnamese employees to assess cross-national differences in profile membership proportions. Between-country differences were tested using chi-square tests (χ^2^) with Cramer’s V as an effect size indicator. Next, we examined associations between profile membership and key sociodemographic variables, including gender (male vs. female), job seniority (in years), job position (entry-level, specialist, managerial), and employment sector (public vs. private). For categorical predictors (gender, position, sector), cross-tabulation analyses with chi-square tests were performed. For continuous variables (seniority), Welch’s ANOVA and Games–Howell *post hoc* tests were applied.

All analyses were conducted in Mplus 8.10 (Muthén and Muthén, 1998–2023) using reproducible syntax, and replicated in R (version 4.4.1; R Core Team, 2024) with the use of relevant packages.

Although no formal *a priori* power analysis was conducted, the target sample size was determined based on methodological recommendations for latent profile analysis and multigroup confirmatory factor analysis. Prior research suggests that mixture models require relatively large samples to ensure stable parameter estimation and reliable class enumeration. Simulation studies (e.g., Nylund et al., 2007) and subsequent methodological reviews indicate that sample sizes of at least 300 are typically recommended for latent profile analysis. The obtained sample sizes in both countries (*N* = 358 and *N* = 305) meet these recommendations and are considered adequate for the planned analyses.

## 4 Results

### 4.1 Preliminary measurement checks

Across all instruments, configural invariance was established, confirming comparable factor structures in Poland and Vietnam. Constraining factor loadings (metric invariance) produced only trivial changes in fit, supporting equality of loadings across countries. Imposing full scalar invariance degraded fit for each scale; however, partial scalar solutions (freeing a small subset of noninvariant intercepts identified via modification indices) restored acceptable fit.

In sum, with metric invariance confirmed and defensible partial scalar models in place, cross-national comparisons of latent covariances and (partially) latent means are warranted, with attention to the freed noninvariant indicators (see Table 2).

**Table 2:** Cross-national (Poland–Vietnam) measurement invariance results for the study measures.

Tool/model	Measurement invariance level	CFI	RMSEA	ΔCFI	ΔRMSEA
PNE/2-factor: PosAtt + NegAtt	Configural	0.958	0.057	—	—
	Metric	0.952	0.059	0.006	0.002
	Partial scalar (items: 1, 4, 8, 10)	0.946	0.061	0.006	0.002
	Scalar	0.909	0.078	0.043	0.021
SAT-20/4-factor: SatDev + SatSal + SatTas + SatRel	Configural	0.900	0.072	—	—
	Metric	0.895	0.073	0.005	0.001
	Partial scalar (items: 5, 7, 10, 14, 20)	0.890	0.073	0.005	0.000
	Scalar	0.875	0.077	0.020	0.004
UWES-9/unidimensional	Configural	0.944	0.088	—	—
	Metric	0.932	0.091	0.012	0.003
	Partial scalar (item 3)	0.922	0.092	0.010	0.001
	Scalar	0.913	0.096	0.021	0.005
DUWAS / 2-factor: ExcWork + ComWork	Configural	0.932	0.068	—	—
	Metric	0.922	0.069	0.010	0.001
	Partial scalar (items: 2, 3, 4, 6)	0.914	0.071	0.008	0.002
	Scalar	0.872	0.084	0.050	0.015

As can be seen in Table 3, internal consistency was generally high in both samples. In Poland/Vietnam, *ω* ranged from 0.78 to 0.98 and *α* from 0.66 to 0.97. Most constructs showed good–excellent reliability (e.g., work engagement: α = 0.91/0.97; ω = 0.94/0.98; satisfaction facets mostly α ≥ 0.82, ω ≥ 0.88). Two exceptions merit note: satisfaction with salary had lower α in Vietnam (0.79, though ω = 0.88), and working compulsively had lower α in Poland (0.66; ω = 0.78). These results support score precision overall, with modest caution for those two scales.

**Table 3:** Internal consistency by country (Poland–Vietnam): Cronbach’s α and McDonald’s ω for all study constructs.

Variable	Poland		Vietnam	
	α	ω	α	ω
Positive affective orientation toward the organization	0.89	0.92	0.88	0.91
Negative affective orientation toward the organization	0.84	0.89	0.89	0.92
Satisfaction with development	0.91	0.92	0.89	0.91
Satisfaction with salary	0.85	0.92	0.79	0.88
Satisfaction with tasks	0.82	0.88	0.85	0.89
Satisfaction with relationships	0.88	0.91	0.86	0.88
Work engagement	0.91	0.94	0.97	0.98
Working excessively	0.78	0.81	0.80	0.83
Working compulsively	0.66	0.78	0.72	0.78

### 4.2 Pooled-sample latent profile analysis

We estimated Gaussian mixture models on the pooled sample (*N* = 663), increasing K from 1 upward and using robust MLR with extensive random starts to avoid local optima. Within-class variances and covariances of the nine indicators were freely estimated but constrained to be equal across profiles (i.e., class separation is driven by means). Model selection balanced information criteria (BIC/SABIC), the BLRT/LMR when applicable, entropy, minimum class size, and substantive interpretability (Wang and Hanges, 2011; Morin and Marsh, 2015).

Fit improved from K = 2 to K = 3 (ΔBIC = 62.6; BLRT *p* < 0.001), yielding three well-separated profiles (entropy = 0.863) with an acceptable smallest class (7.8%). Moving to K = 4 further lowered BIC/SABIC (ΔBIC = 18.4; BLRT p < 0.001) and slightly increased entropy (0.878), but produced a very small class (3.5%). Inspection suggested that the fourth class partitioned an existing group without adding a substantively novel configuration, raising concerns about stability (replicability) for such a small subgroup. Balancing parsimony, separation, and class size adequacy, we retained K = 3 as the optimal working solution for subsequent cross-national profile-similarity tests (Table 4).

**Table 4:** Model comparison for pooled-sample latent profile solutions (K = 2–4): fit indices, class proportions, and classification quality (*N* = 663).

K	LogLik	#Par	AIC	BIC	SABIC	Entropy	Class proportions	BLRT vs K-1
2	−6947.12	64	14022.24	14310.04	14106.83	0.875	0.129/0.871 (min = 0.129)	*p* < 0.001 (1 → 2)
3	−6883.33	74	13914.66	14247.42	14012.47	0.863	0.252/0.078/0.670 (min = 0.078)	*p* < 0.001 (2 → 3)
4	−6841.67	84	13851.33	14229.06	13962.36	0.878	0.035/0.075/0.670/0.220 (min = 0.035)	*p* < 0.001 (3 → 4)

K, number of latent profiles (classes) in the model; LogLik, model log-likelihood for the fitted solution at K (higher, i.e., less negative, indicates better fit); #Par, number of freely estimated parameters; AIC, Akaike Information Criterion; BIC, Bayesian Information Criterion; SABIC, Sample-size adjusted BIC (lower AIC/BIC/SABIC indicate better fit); Entropy, classification quality index ≥ 0.80 indicates good classification; Class proportions – estimated class shares; the value in parentheses is the smallest share (classes < 5% are considered unstable in applied profiling); BLRT vs K − 1 – parametric Bootstrap Likelihood Ratio Test comparing the K-class model to the (K − 1)-class model; *p* < 0.05 supports the K-class solution (requires robust random starts).

**Figure 1:**
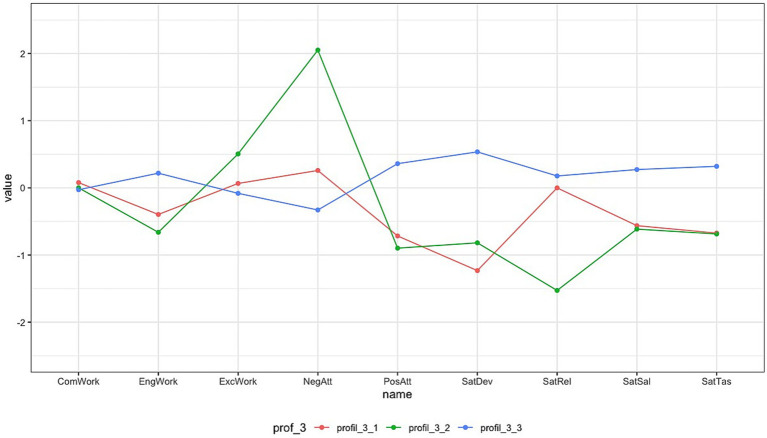
Standardized means of nine indicators across the three identified profiles (pooled sample, *K* = 3). ComWork = working compulsively, EngWork = work engagement, ExcWork = working excessively, NegAtt = negative affective orientation toward the organization, PosAtt = positive affective orientation toward the organization, SatDev = satisfaction with development, SatSal = satisfaction with salary, SatRel = satisfaction with relationships, SatTas = satisfaction with tasks.

To sum up, the three-profile solution provides a good compromise between statistical fit and practical usefulness: high classification quality, no extremely small class, and clear separation via profile means under a common covariance structure. We therefore use K = 3 (see Figure 1) as the reference structure for multigroup (Poland vs. Vietnam) profile-similarity analyses (configural, structural, dispersion, distributional), and then proceed to covariate/distal modeling.

### 4.3 Between-profile differences on standardized indicators

To further validate the distinctiveness of the latent profiles, we compared the standardized means of the nine indicators across profiles using Welch’s ANOVAs with Games–Howell *post hoc* tests (see Table 5 for full results). Significant differences were observed for eight of the nine indicators, with the exception of working compulsively.

**Table 5:** Between-profile differences on standardized indicators (based on Welch ANOVA and Games–Howell tests).

Variable	F (df1, df2)	*p*	Significant pairwise differences (Games–Howell, *p* < 0.05)	Pattern of means
Working compulsively (ComWork)	0.60 (2, 134.92)	0.55	—	—
Work engagement (EngWork)	34.43 (2, 124.41)	< 0.001	P3 > P1, P3 > P2	P3 > others
Working excessively (ExcWork)	11.45 (2, 137.60)	< 0.001	P1 < P2, P2 > P3	P2 > P3 > P1
Negative affective orientation (NegAtt)	196.81 (2, 120.68)	< 0.001	P2 > P1 > P3	P2 > P1 > P3
Positive affective orientation (PosAtt)	106.09 (2, 121.77)	< 0.001	P3 > P1, P3 > P2	P3 > others
Satisfaction with development (SatDev)	540.14 (2, 126.33)	< 0.001	P3 > P1, P3 > P2, P1 > P2	P3 > P1 > P2
Satisfaction with relationships (SatRel)	47.30 (2, 121.76)	< 0.001	P3 > P2, P1 > P2	P2 < others
Satisfaction with salary (SatSal)	54.68 (2, 124.51)	< 0.001	P3 > P1, P3 > P2	P3 > others
Satisfaction with tasks (SatTas)	74.23 (2, 118.11)	< 0.001	P3 > P1, P3 > P2	P3 > others

P1 = moderately disengaged; P2 = overworked and discontent; P3 = engaged and satisfied. Welch’s ANOVA and Games–Howell tests were used. Values are based on standardized indicators.

*Post hoc* comparisons revealed a consistent pattern of differences. The Engaged and satisfied profile (Profile 3) scored significantly higher than the other two profiles on work engagement, positive affective orientation, and all facets of job satisfaction (development, pay, relationships, and tasks; all ps < 0.001). The Overworked and discontent profile (Profile 2) showed the highest negative affective orientation, significantly exceeding both the Moderately disengaged and Engaged profiles (*p* < 0.001), while also reporting the lowest satisfaction across job facets. In contrast, the Moderately Disengaged profile (Profile 1) exhibited midrange scores: average working excessively and compulsively, moderate negative affect, and below-average positive affect and satisfaction levels. Differences in working compulsively were nonsignificant across profiles.

Taken together, these results confirm that the three latent profiles represent conceptually coherent and statistically distinct constellations of work attitudes and behaviors: (a) Moderately Disengaged – lower engagement and satisfaction but not overtly negative; (b) Overworked and Discontent – high strain and dissatisfaction; and (c) Engaged and Satisfied – highly engaged, positive, and content employees.

### 4.4 Multigroup LPA: profile similarity across countries

A three-profile multigroup LPA with country as a known class converged with a replicated best log-likelihood and good classification. The configural model (same number of profiles in Poland and Vietnam, all class-specific parameters free; M0) fit best among the tested invariance models (LL = −7390.29; AIC = 15002.57; BIC = 15501.71; SABIC = 15149.28; entropy = 0.90). Pooled class prevalences were 43.4, 22.0, and 34.6% (N_Poland_ = 358; N_Vietnam_ = 305), with adequate separation.

**Figure 2:**
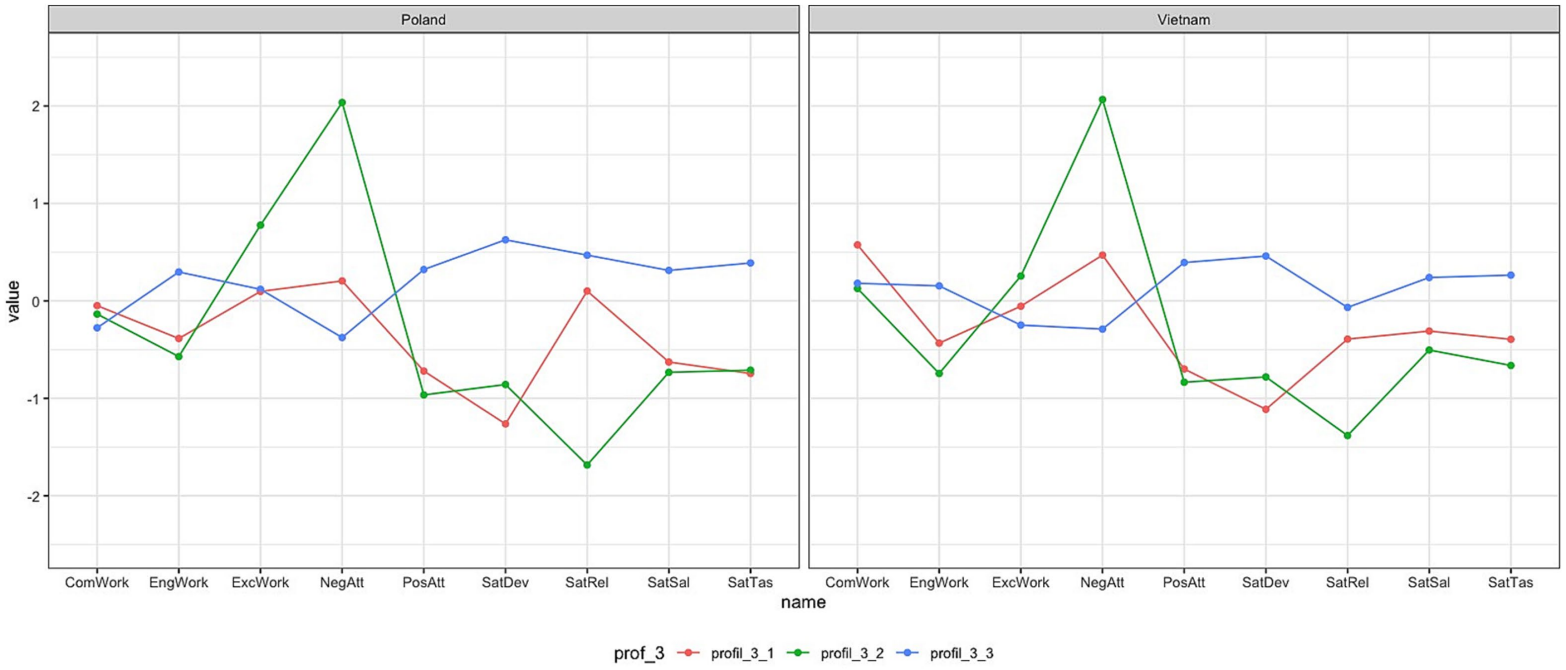
Standardized means of the three identified profiles by country: Poland (left panel) and Vietnam (right panel). ComWork = working compulsively, EngWork = work engagement, ExcWork = working excessively, NegAtt = negative affective orientation toward the organization, PosAtt = positive affective orientation toward the organization, SatDev = satisfaction with development, SatSal = satisfaction with salary, SatRel = satisfaction with relationships, SatTas = satisfaction with tasks.

Structural (shape) similarity (which constrains indicator means to be equal across corresponding profiles in Poland and Vietnam; M1) fit worse than the configural model (M0; BIC = 15630.56 vs. 15501.71; ΔBIC = +128.85). This indicates that some profile means differ by country. Importantly, the qualitative shapes of the profiles were the same in both countries (see Figure 2): (1) Latent Profile 1 – Moderately disengaged; (2) Latent Profile 2 – Overworked and discontent; and (3) Latent Profile 3 – Engaged and satisfied. Thus, we retain the less restrictive model and interpret the same three shapes across countries, acknowledging country differences in levels.

Adding dispersion (scale) similarity (equality of within-profile variances across countries; M2) further degraded fit (LL = −7642.88; BIC = 15656.07; ΔBIC vs. M1 = +25.51), arguing against equal residual dispersion by country.

Finally, the distributional similarity model (equal class proportions; M3) yielded the same log-likelihood as M2 (LL = −7642.88). This equivalence arises because, under the current parameterization with a known grouping variable, class proportions were already held common across countries in the preceding models; consequently, M3 did not introduce an additional test.

To conclude, the multigroup analysis supports configural similarity (the same number and qualitative shapes of profiles in Poland and Vietnam) but not structural or dispersion similarity. We therefore retain the three-profile solution with country-specific means and variances, and we compare countries descriptively on profile prevalences and indicator levels within profiles (illustrated in Figure 2).

### 4.5 Profile prevalence analysis

A chi-square test of independence indicated that the distribution of work attitude profiles differed significantly between Polish and Vietnamese employees, χ^2^(2, 663) = 55.80, *p* < 0.001. The effect size, measured by Cramer’s V, was moderate (V = 0.29, 95% CI [0.22, 1.00]). Specifically, Profile 3 (Engaged and satisfied) was more prevalent in the Vietnamese sample (80%) than in the Polish sample (57%), whereas Profile 1 (Moderately disengaged) was more common in Poland (36%) than in Vietnam (11%).

A chi-square test of independence showed that profile membership did not differ by gender, χ^2^(2, 663) = 0.86, *p* = 0.65, V < 0.01. A significant association emerged between job position and profile membership, χ^2^ (4, 663) = 19.40, *p* < 0.001, V = 0.11, 95% CI [0.03, 1.00], indicating that managerial employees were overrepresented in Profile 3 (Engaged and satisfied) compared with entry-level employees. No significant association was found between employment sector and profile membership, χ^2^ (2, 663) = 1.51, *p* = 0.47, V < 0.01.

A Welch’s ANOVA revealed significant differences in job seniority across work attitude profiles, *F* (2, 131) = 5.25, *p* = 0.006. *Post hoc* Games–Howell tests showed that employees in Profile 3 (Engaged and satisfied) had significantly longer job tenure (M = 13.5, SD = 8.30) compared with those in Profile 1 (Moderately disengaged; M = 11.7, SD = 7.96; ΔM = 1.77, 95% CI [0.03, 3.51], *p* = 0.045, g = −0.22, 95% CI [−0.40, −0.04]) and Profile 2 (Overworked and discontent; ΔM = 3.14, 95% CI [0.20, 6.07], *p* = 0.034, g = −0.37, 95% CI [−0.66, −0.08]). The difference between Profiles 1 and 2 was nonsignificant (ΔM = −1.36, *p* = 0.56, g = 0.17). Overall, employees in Profile 3 exhibited longer tenure than those in the other two profiles, suggesting that more experienced workers were more likely to display the attitudinal pattern represented by Profile 3.

## 5 Discussion

### 5.1 Summary of main findings

This cross-national study identified three qualitatively distinct constellations of work attitudes (Moderately disengaged, Overworked & discontent, and Engaged & satisfied) by integrating affective orientations, four job-satisfaction facets, engagement, and workaholic tendencies within a person-centered framework. Preliminary psychometrics supported configural and metric invariance and defensible partial scalar invariance for all instruments across Poland and Vietnam, enabling comparisons of latent covariances and cautiously of latent means. In pooled analyses, the three-profile solution offered the best balance of fit, parsimony, and class adequacy, with clear between-profile differences on eight of nine indicators (no differences for working compulsively). Multigroup LPA supported configural similarity but not structural or dispersion similarity, indicating that the profile shapes replicated across countries while levels and within-profile variances differed. Profile prevalence varied by country (Engaged & satisfied more common in Vietnam; moderately disengaged more common in Poland), whereas gender and sector were unrelated to membership; managers and employees with longer tenure were overrepresented in the Engaged & satisfied profile.

### 5.2 Theoretical contributions

First, by simultaneously modeling affective (positive/negative organizational orientations), cognitive–evaluative (four satisfaction facets), and motivational–behavioral (engagement vs. working excessively/compulsively) dimensions, our person-centered approach demonstrates that employee functioning organizes into coherent typologies rather than varying only along single continua. This advances person-centered scholarship by moving beyond variable-centered associations to reveal configurations that are theoretically interpretable and empirically distinct (Wang and Hanges, 2011; Morin and Marsh, 2015; Morin et al., 2018). In doing so, the study clarifies that patterns of attitudes and behaviors are emergent properties of the work system, captured only when the attitudinal space is modeled holistically.

Second, the content of the profiles elaborates core mechanisms posited by JD–R, AET, and SDT. The Engaged & Satisfied profile maps onto a resource-rich, need-supportive ecology in JD–R and SDT terms, where autonomy, growth, and social support are likely to satisfy basic psychological needs and sustain positive affect (Bakker and Demerouti, 2017; Deci and Ryan, 2000). Conversely, the Overworked & Discontent profile reflects high demands and strain, consistent with JD–R’s health-impairment pathway and AET’s prediction that accumulations of adverse events consolidate into enduring negative orientations (Weiss and Cropanzano, 1996). The Moderately Disengaged profile occupies a meso-zone with muted positive affect and satisfaction, suggesting contexts where resources are present but insufficiently need-supportive to catalyze vigorous engagement. Together, these findings integrate JD–R’s resource/demand dynamics with AET’s affect accumulation and SDT’s need fulfillment by showing that their joint operation yields stable, person-level configurations rather than isolated effects.

Third, the cross-cultural evidence contributes to theory on universality versus contextuality. Configural similarity across Poland and Vietnam indicates a shared taxonomy of work-attitude patterns, aligning with recent work showing that person-centered structures in work motivation generalize across cultures (Woo et al., 2024). At the same time, differences in mean levels and prevalence (most notably the higher incidence of Engaged & satisfied in Vietnam) point to contextual modulation consistent with documented differences in work values and expectations among Vietnamese employees (Tran et al., 2017) and with institutional/cultural distinctions discussed in our framework. Theoretically, this combination of form invariance (shared shapes) and level variability refines cross-cultural models by specifying which aspects of work attitudes are etic (structural) and which are emic (distributional/mean-level).

Fourth, the null profile differences for compulsive work (amid clear differences for engagement and excessive work) sharpen theory on the engagement–workaholism nexus. Rather than constituting opposite ends of a single continuum, engagement (energetic, self-endorsed) and workaholism (pressure-driven) can co-reside within profiles in distinct ways, corroborating and extending arguments that these are partially orthogonal constructs (Schaufeli et al., 2008). This nuance encourages theorists to move from simple antagonistic models toward multidimensional accounts that differentiate excessive (behavioral) from compulsive (cognitive) investment and consider how each aligns with affective tone and satisfaction within real-world constellations.

Fifth, by linking profile membership to role seniority and managerial status, the study points to developmental and structural processes through which resources, affective experiences, and need fulfillment accumulate over time to stabilize adaptive configurations. This observation invites theory to incorporate temporal pathways (how employees transition between configurations as demands, resources, and need support evolve) thus providing a bridge from static typologies to dynamic models of profile mobility.

Sixth, the findings refine the theoretical role of sociodemographic characteristics in person-centered models of work attitudes. Consistent with resource-based perspectives, employees in managerial positions and those with longer tenure were more likely to belong to the Engaged & satisfied profile, suggesting that access to resources and accumulated experience supports more adaptive attitudinal configurations. In contrast, the absence of differences by gender and employment sector indicates that these factors may play a more limited or context-dependent role in shaping holistic patterns, even if they remain relevant for specific work behaviors. These results suggest that structural position and experience, rather than demographic category per se, are more central for understanding how multidimensional work attitudes coalesce at the person level.

### 5.3 Practical implications

The present findings offer several actionable directions for organizations seeking to enhance employee well-being and performance through evidence-based, contextually grounded interventions.

The identification of three distinct constellations of work attitudes underscores the importance of tailoring organizational interventions to the needs of each subgroup rather than relying on one-size-fits-all approaches: (1) Moderately disengaged: interventions should focus on job redesign (increasing task significance, variety, and autonomy) to restore psychological meaningfulness and stimulate intrinsic motivation. Structured career development pathways and recognition systems can further enhance positive affect and satisfaction by signaling fairness and value alignment. (2) Overworked & discontent: this group would benefit from reducing excessive job demands through workload calibration and boundary management initiatives, complemented by recovery-promoting resources such as rest breaks and supportive supervision. Efforts to improve managerial fairness and team climate are also critical, as these employees often experience high strain and low perceived support. (3) Engaged & satisfied: organizations should aim to sustain resource gains by maintaining autonomy, growth opportunities, and strong social support systems. However, given the potential for high engagement to slide into overcommitment, regular monitoring of workload and recovery is advised to prevent transitions toward maladaptive overwork.

Cross-national differences in profile prevalence and mean levels suggest that interventions must be sensitive to cultural and institutional contexts. In Vietnam, emphasizing relational support, collective goals, and long-term development pathways may resonate more with prevailing collectivist and harmony-oriented norms. In contrast, in Poland, initiatives promoting clear procedures, fairness, and role clarity may be more effective, aligning with higher uncertainty avoidance and a stronger emphasis on procedural justice (Hofstede et al., 2010; Tran et al., 2017). Thus, culturally responsive HR practices can strengthen the alignment between organizational resources and employees’ motivational orientations.

The overrepresentation of managers and long-tenure employees within the Engaged & satisfied profile highlights the value of leadership development and retention-focused strategies in fostering adaptive constellations of work attitudes. By contrast, early-career employees are more likely to fall into the moderately disengaged group, suggesting that onboarding programs, mentorship, and early support mechanisms can serve as preventive interventions against disengagement. Leadership behaviors that model healthy engagement (demonstrating enthusiasm while maintaining boundaries) can also buffer subordinates from the pressures that often lead to overwork.

## 6 Limitations and future research

While the study contributes novel insights, several limitations should guide interpretation and future inquiry. First, the cross-sectional design limits causal inference. Longitudinal person-centered analyses (tracking transitions between profiles over time) could determine whether shifts in job demands, resources, or need support predict profile mobility. Such dynamic modeling would capture how employees evolve along the engagement–disengagement continuum as their work conditions and experiences change.

Second, both national samples comprised formally employed, salaried workers in established organizations. This scope excluded self-employed individuals, platform workers, informal laborers, and those contributing to family businesses. Including these groups in future research would introduce greater heterogeneity and offer a more realistic representation of the employment landscape in both countries. Such workers often experience different constellations of autonomy, insecurity, and social support – conditions that could generate new or hybrid profiles (e.g., highly engaged yet precariously situated, or relationally committed despite limited formal resources). Expanding the sampling frame would thus enhance the ecological validity and cultural representativeness of the profile taxonomy.

Third, although partial scalar invariance was established, this solution still warrants caution when interpreting cross-national latent mean differences. Future studies could employ alignment optimization to test robustness. Further, the nine-indicator structure (though conceptually comprehensive) captures only a subset of the broader work-attitude space. Including constructs such as organizational identification, perceived justice, or recovery experiences may reveal more differentiated subtypes and clarify boundary conditions of existing profiles.

Fourth, the present cross-cultural design compared two institutional contexts (Poland and Vietnam) that differ in governance, economic structure, and value systems. Replication across additional regions with varying labor-market regimes would enable stronger tests of cultural moderators (e.g., power distance, uncertainty avoidance) and strengthen claims about universality versus specificity in work-attitude patterns (Woo et al., 2024).

Finally, the absence of significant profile differences in compulsive work invites theoretical refinement. Future studies could integrate multidimensional or network approaches to examine how cognitive and affective components of workaholism interact with engagement. Combining experience sampling (to capture affective events per AET) and physiological or behavioral indicators of recovery could elucidate when and how compulsive tendencies detach from adaptive engagement.

## 7 Conclusion

The study uncovers a replicable, three-profile structure of multidimensional work attitudes that bridges affective experience, evaluative judgments, and motivational-behavioral investment. Profiles replicate across Poland and Vietnam at the configural level while differing in levels and dispersion, underscoring both universality and context. Integrating JD–R, AET, and SDT within a person-centered lens advances theory on how demands/resources, affective events, and need fulfillment crystallize into actionable employee segments. Practically, the profiles provide a diagnostic map for tailoring interventions to local contexts and workforce strata. Future longitudinal and cross-cultural extensions can test causal mechanisms, refine invariance, and connect profiles to hard outcomes, moving from description to evidence-based profile management.

## Data Availability

The datasets presented in this study can be found in online repositories. The names of the repository/repositories and accession number(s) can be found at: https://osf.io/emy4s/overview?view_only=67ba35ef1fad4c87a51298efc3b2b74c.
